# Scarlet Fever

**Published:** 1876-03

**Authors:** E. H. Gibbs


### SCARLET FEVER.

BY E. H. GIBBS, A.M., M.D.

No disease that children are liable to is so much feared as "scarlet fever." A half century ago "small pox" was the horror of horrors, but Jenner divested it of its terrors. More recently "cholera infantum" has headed the list of fatal infantile diseases. Now there is no disease that yields more promptly and more certainly to proper treatment. "Scarlet fever" assumes so many forms, and is liable to so many complications, that it would seem charlatanical to suggest a treatment that would apply to all cases, as we could in the other diseases above referred to. In the first, proper vaccination is a certain preventive of small pox, and in "cholera infantum," and all bowel complaints of children, the proper remedy in the hands of the mother or nurse is just as certainly curative. But in case of scarlet fever, the physician should always be called to determine the type of the disease, and to watch its course and progress.

It is contagious, and is, probably, always disseminated in this way; still, a child in perfect health, and with the system toned up by proper nourishment and cleanliness, may be frequently exposed to the disease without contracting it. It is far more prudent, however, to protect children from such exposure. It may save many sad regrets afterward. When a child passes its eighth year its liability to contract the disease steadily diminishes, and at fifteen years it enjoys almost entire immunity from it.

In the number of this JOURNAL for July, 1875, I fully described SCARLET FEVER, and the diseases with which it is liable to be confounded. What parents, and all other humane persons are interested in knowing is, the best and safest treatment. Eleven years ago last August I received a telegram from a medical friend in a neighboring city stating that scarlet fever of the severest form was making sad havoc among the children, and asking me to come to his assistance. Responding promptly to the call, a journey of about four hours brought me to a community that were fairly "panic-stricken." The disease was very prevalent and alarmingly fatal. We visited houses where all the children in the family—in some cases three or four—were sick.

In such emergencies time is precious, and we set ourselves to work promptly and actively. In all recent cases, where the eruption had not come out freely, warm baths were ordered, and to be repeated every five hours, until a free eruption appeared. In cases of great debility sponge baths were used, or cloths dipped in hot water and wrung as dry as possible. It is well in all cases to alternate these with the baths. In cases of longer standing, where the eruption had appeared, but the skin was dry and parched, we *invariably* smeared the little patient over the entire body, using a piece of fat bacon or a piece of fat cut from a smoked ham.

An educated physician long ago recommended this remedy. I have always employed it since that time in every case I have treated. My medical friend lost no more little patients, and he declared to the day of his death that the "bacon saved them."

Childhood should be the least hazardous period of human existence. That it is not, is the fault of those who are intrusted with the care of children—a fault that arises either from neglect or inexperience. Not one child in a hundred, suffering from any of the diseases incident to childhood, would die were it properly cared for. Young children, whether sick or well, suffer more from thirst than from any other single cause. A little cold water should be given to a child every hour in the day from the time it is a day old until it can make its wants known. In all cases of sickness, from whatever cause, little pieces of ice may be put into the mouth at short intervals, which, in dissolving, will allay irritation of the mouth and throat and quench the thirst. There is no remedy equal to it for diptheria and other diseases of the mouth and throat.

